# Severity of Old World Cutaneous Leishmaniasis Is Influenced by Previous Exposure to Sandfly Bites in Saudi Arabia

**DOI:** 10.1371/journal.pntd.0003449

**Published:** 2015-02-03

**Authors:** Karina Mondragon-Shem, Waleed S. Al-Salem, Louise Kelly-Hope, Maha Abdeladhim, Mohammed H. Al-Zahrani, Jesus G. Valenzuela, Alvaro Acosta-Serrano

**Affiliations:** 1 Department of Parasitology, Liverpool School of Tropical Medicine, Liverpool, United Kingdom; 2 Department of Vector Borne Diseases, Saudi Ministry of Health, Riyadh, Kingdom of Saudi Arabia; 3 Centre for Neglected Tropical Diseases, Liverpool School of Tropical Medicine, Liverpool, United Kingdom; 4 Vector Molecular Biology Section, National Institute of Allergy and Infectious Diseases, National Institutes of Health, Bethesda, Maryland, United States of America; 5 Department of Vector Biology, Liverpool School of Tropical Medicine, Liverpool, United Kingdom; Makerere University, Uganda

## Abstract

**Background:**

The sandfly *Phlebotomus papatasi* is the vector of *Leishmania major*, the main causative agent of Old World cutaneous leishmaniasis (CL) in Saudi Arabia. Sandflies inject saliva while feeding and the salivary protein PpSP32 was previously shown to be a biomarker for bite exposure. Here we used recombinant PpSP32 to evaluate human exposure to *Ph. papatasi* bites, and study the association between antibody response to saliva and CL in endemic areas in Saudi Arabia.

**Methodology/Principal Findings:**

In this observational study, anti-PpSP32 antibodies, as indicators of exposure to sandfly bites, were measured in sera from healthy individuals and patients from endemic regions in Saudi Arabia with active and cured CL. *Ph. papatasi* was identified as the primary CL vector in the study area. Anti-PpSP32 antibody levels were significantly higher in CL patients presenting active infections from all geographical regions compared to CL cured and healthy individuals. Furthermore, higher anti-PpSP32 antibody levels correlated with the prevalence and type of CL lesions (nodular vs. papular) observed in patients, especially non-local construction workers.

**Conclusions:**

Our findings suggest a possible correlation between the type of immunity generated by the exposure to sandfly bites and disease outcome.

## Author Summary

*Leishmania* is transmitted by the bite of infected female sandflies. When a sandfly bites a vertebrate host, it injects a cocktail of salivary proteins meant to facilitate blood feeding. The constant exposure to sandfly bites in endemic areas triggers a humoral response against the major antigenic components in the saliva. These antibodies can be then exploited to measure exposure to vector sandflies, which is useful for surveillance in leishmaniasis control programmes. In Saudi Arabia, cutaneous leishmaniasis (CL) is mainly transmitted by the *Phlebotomus papatasi* sandfly. Here we study the recognition of the main antigenic salivary protein from *Ph. papatasi*, PpSP32, in leishmaniasis patients and healthy individuals from three CL endemic areas in Saudi Arabia. Anti-PpSP32 antibody levels were significantly higher in CL patients presenting active infections from all geographical regions compared to the CL-cured and healthy individuals. Furthermore, higher anti-PpSP32 antibody levels correlated with the prevalence and type of CL lesions observed in patients. Our results suggest that previous long-term exposure to sandfly saliva can have a role in modulating the severity of leishmaniasis infection, resulting in a milder form of the disease.

## Introduction

Cutaneous leishmaniasis (CL) in Saudi Arabia is an increasing public health problem due to rapid urbanization, intensive agriculture and human migration [1]. Zoonotic CL (ZCL) is the most prevalent form of leishmaniasis in the country, which is caused by *Leishmania major* and transmitted by the sandfly *Phlebotomus papatasi. Leishmania tropica* on the other hand is exclusively endemic to the South Western region [2], where it is transmitted by *Ph. sergenti* and causes anthroponotic CL (ACL).

The saliva that sandflies inject into their vertebrate host impairs the haemostatic and inflammatory systems allowing the insects to efficiently take a blood meal [3]. These salivary components were also shown to promote or inhibit the development of *Leishmania* in the vertebrate host [4]. Increased sandfly-host contact translates into an increased risk of being infected. Repeated exposure to sandfly bites produces antibodies against its salivary components in the host, providing an indirect measure of exposure to vectors [5]. The presence of IgG antibodies against *Ph. papatasi* saliva has been associated with a higher risk of being infected with *L. major* [4,6]. The transient nature of the antibody response to sandfly bites [6–10] allows for the study of temporal changes in transmission risk and the efficacy of vector control programmes [11].

Biomarkers used to evaluate sandfly exposure need to be species-specific in order to differentiate between antibody responses to vector and non-vector species, or between sandflies and other blood-feeding insects including mosquitoes. The sandfly salivary protein PpSP32 has been described as a 30 kDa immunodominant target of the host antibody response against *Ph. papatasi* saliva [12,13], and was highly specific when tested against individuals living in a region with high prevalence of *Ph. perniciosus*. Additionally, expression of the *PpSP32* salivary transcript is not influenced by age or diet of the sandfly [14]. B-cell epitope prediction analysis showed six epitopes were identical between the Tunisian PpSP32 and the PpSP32 protein deposited in GenBank (Israeli strain), indicating it is a good candidate to assess biting exposure in different ZCL foci [13]. Furthermore, the production of rPpSP32, a recombinant form of the *Ph. papatasi* PpSP32 protein, overcomes the difficulty of obtaining large quantities of salivary glands, and facilitates the use of salivary biomarkers for large scale epidemiological studies in endemic areas.

To better understand the correlation between sandfly biting exposure and leishmaniasis infection, we determined the level of exposure to *Ph. papatasi* bites in individuals from several CL endemic areas in Saudi Arabia by measuring the levels of anti-PpSP32 antibodies present in the sera of patients and healthy volunteers.

## Materials and Methods

### Ethics statement

The study was approved by the Liverpool School of Tropical Medicine Ethics Committee UK (12.03RS). All participants provided written informed consent for the collection of blood samples and subsequent analyses. All research was conducted according to Declaration of Helsinki principles.

### Study samples

Peripheral blood samples were obtained from 411 individuals (106 females and 305 males, aged 18–60 years, median of 36 years) living in two ZCL (Al Ahsa and Al Madinah) and one ACL (Asir) endemic areas in Saudi Arabia (S1 Table). Study sites were chosen to include areas were patients would be exposed to the bite of *Ph. papatasi* (ZCL transmission) or *Ph. sergenti* (ACL transmission) (Al Salem et al, 2014. *Submitted*) to test the specificity of the biomarker. Samples were collected during the months of April and December 2012. Cases were diagnosed through parasitological confirmation of *Leishmania* by a trained clinician, and the infecting *Leishmania* species was confirmed in patients with both active and cured infections (through clinical history). Clinical cure was signified by successful re-epithelialisation of the lesion(s) after treatment.

Donor sera were classified as healthy (no history of leishmaniasis infection), ZCL (*L. major*) or ACL (*L. tropica*) patients with either active or cured CL. An additional 80 serum samples of patients with active infection from Al Ahsa were used for the analysis of local versus non-local exposure; although these were likely to be infections with *L. major*, they are unconfirmed and therefore considered separately. We used sera from five United Kingdom residents as non-endemic controls. These healthy volunteer donors have no history of leishmaniasis or travelling to sandfly endemic areas.

### Expression and purification of PpSP32 recombinant protein

Mammalian VR-2001 plasmid coding the PpSP32 protein with 6 histidine tag was sent to the Protein Expression Laboratory at the Frederick National Laboratory for Cancer Research (Frederick, Maryland). Expression was performed by transfecting HEK-293F cells. The supernatant was collected after 72 hours, filtered and concentrated from 1 litre to 300 ml using an Amicon concentrator device (Millipore, Billerica, MA, USA) in the presence of NaCl 500mM. The volume was returned to 1 litre at a final concentration of 10 mM Tris, pH 8.0. The expressed protein was purified by an HPLC system (DIONEX, CA, USA) using two 5 ml HiTrap Chelating HP columns (GE Healthcare, Buckinghamshire, UK) in tandem and charged with 0.1 M NiSO4. The protein was detected at 280 nm and eluted by an imidazole gradient as described by Teixeira et al. [15]. Eluted proteins were collected every minute in a 96-well microtiter plate using a Foxy 200 fraction collector (Teledyne ISCO, Lincoln, NE, USA). Fractions corresponding to eluted proteins peaks were selected and run on a NuPage Bis-Tris 4–12% Gel (Novex, Life Technologies, Carlsbad, CA, USA) with MES running buffer under reducing conditions as per manufacturer’s instructions. Appropriate fractions, as determined by molecular weight were pooled and concentrated to 1 ml using an Amicon Ultra Centrifugal Filter (Millipore, Billerica, MA, USA). Protein concentration was measured using a NanoDrop ND-1000 (Thermo Scientific, Waltham, MA, USA) spectrophotometer at 280 nm and calculated using the extinction coefficient of the protein.

### Detection of human anti-PpSP32 antibodies

Exposure to sandfly bites was measured through the levels of anti-PpSP32 IgG antibodies in the sera of participants. Anti-PpSP32 antibodies were measured by ELISA (Enzyme-Linked Immunosorbent Assay), as described by Marzouki et al. [13] with some modifications. Briefly, microtiter plates (Thermo-Scientific) were coated overnight with 50 μl of PpSP32 (2 mg/ml = 0.1 mg/well) in 0.1M carbonate buffer (pH 9.6). Plates were blocked with PBS-BSA at 37°C for one hour and then washed several times with PBS. Diluted sera (1:200) were added to the plates and incubated at 37°C for 2 hours. After washing, plates were incubated with anti-human IgG peroxidase-conjugated antibody (1:10000) (Jackson ImmunoResearch, Suffolk, UK) for one hour at 37°C. Antibody binding was visualized using the substrate, 3,3′,5,5′ tetramethylbenzidine (Biolegend, San Diego, CA, USA), and absorbance was read at 450 nm on a Fluorostar Omega microplate reader (BMG Labtech, Ortenberg, Germany). Each serum was tested in triplicate. Wells without serum were used as negative controls.

### Sandfly vector species in CL endemic areas

To determine the relative abundance of vector species in each of the endemic areas, sandfly collection was conducted between March and November of 2012. Adult sandflies were collected using CDC light traps placed from 6:00pm to 6:00am in the peridomicile of houses, including sheds harboring domestic animals such as chickens and rabbits. Sticky traps were used to capture sandflies in rodent burrows. Sandflies were preserved in 70% alcohol and identified to species [16].

### Distribution maps

Software ArcGIS 10 (ESRI, Redlands CA) was used to show the presence of vector species.

### Statistical analysis

The Kruskal-Wallis test was used to compare sets of groups. GraphPad Prism Software 5 was used for all data analysis. Statistical significance was considered as *P*<0.05.

## Results

### *Ph. papatasi* was the only vector species found in Al Ahsa and Al Madinah, while in Asir *Ph. sergenti* was the most common

**Figure 1:**
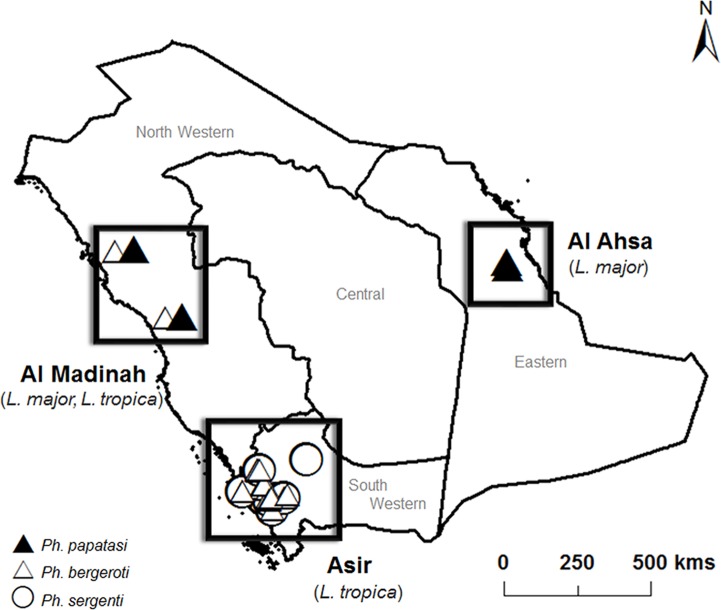
Map of Saudi Arabia indicating the presence of sandfly vector species in several areas endemic for cutaneous leishmaniasis. *Phlebotomus papatasi* is prevalent in Al Ahsa and Al Madinah. In Asir, *Ph. sergenti* is the most common vector species. Symbols are representative of sampling locations and do not reflect species abundance. Filled triangle: *Ph. papatasi*; Open triangle: *Ph. bergeroti*; Open circle: *Ph. sergenti*.

In the regions of Al Ahsa and Al Madinah, ∼99% of sandflies were identified as *Ph. papatasi*, with the additional presence of a few *Ph. bergeroti* (∼1%) in Al Madinah (Table 1). The Southern region of Asir showed the highest diversity of vector species; *Ph. sergenti* was the most abundant (21%), followed by *Ph. bergeroti* (10%). Although *Sergentomyia* species (of non-medical importance) represented only a small percentage (∼1%) in Al Ahsa and Al Madinah, they constituted over half of the specimens identified in Asir (67%). The predominant presence of *Ph. papatasi* in both Al Ahsa and Al Madinah, and of *Ph. sergenti* in Asir, is in agreement with the prevalence of infections caused by *L. major* and *L. tropica*, respectively (Fig. 1).

**Table 1:** Sandfly species in the cutaneous leishmaniasis endemic regions.

**Species**	**Al Ahsa^a^**	**Al Madinah^a^^,^^b^**	**Asir^b^**
*Ph. papatasi*	99%	99%	1%
*Ph. bergeroti*	0	<1%	10%
*Ph. sergenti*	0	0	21%
*Ph. alexandri*	0	0	<1%
*Ph. orientalis*	0	0	<1%
*Sergentomyia* spp.^c^	1%	<1%	67%

Sandflies were collected using CDC light traps around houses of leishmaniasis patients, and sticky traps were used in rodent burrows *Ph. papatasi* was the most abundant species in Al Ahsa and Al Madinah. However, in Asir *Ph. sergenti* was the dominant vector, followed by *Ph. bergeroti*.

^a.^ Region with Zoonotic Cutaneous Leishmaniasis cases

^b.^ Region with Anthroponotic Cutaneous Leishmaniasis cases

^c.^ Genus with no *Leishmania* vector species

### PpSP32 is recognized by sera of individuals living in CL endemic areas of Saudi Arabia where *Ph. papatasi* is prevalent

We found that the levels of anti-PpSP32 antibodies in the sera of healthy individuals from Saudi Arabia were significantly higher (*P*≤0.01) (S1 Fig.) when compared to unexposed individuals from the UK. This indicates the biomarker is successfully recognized by Saudi individuals, and furthermore agrees with the expected level of exposure to sandflies in CL-endemic areas.

### In Al Ahsa and Al Madinah the levels of anti-PpSP32 antibodies are higher in CL patients than healthy individuals

**Figure 2:**
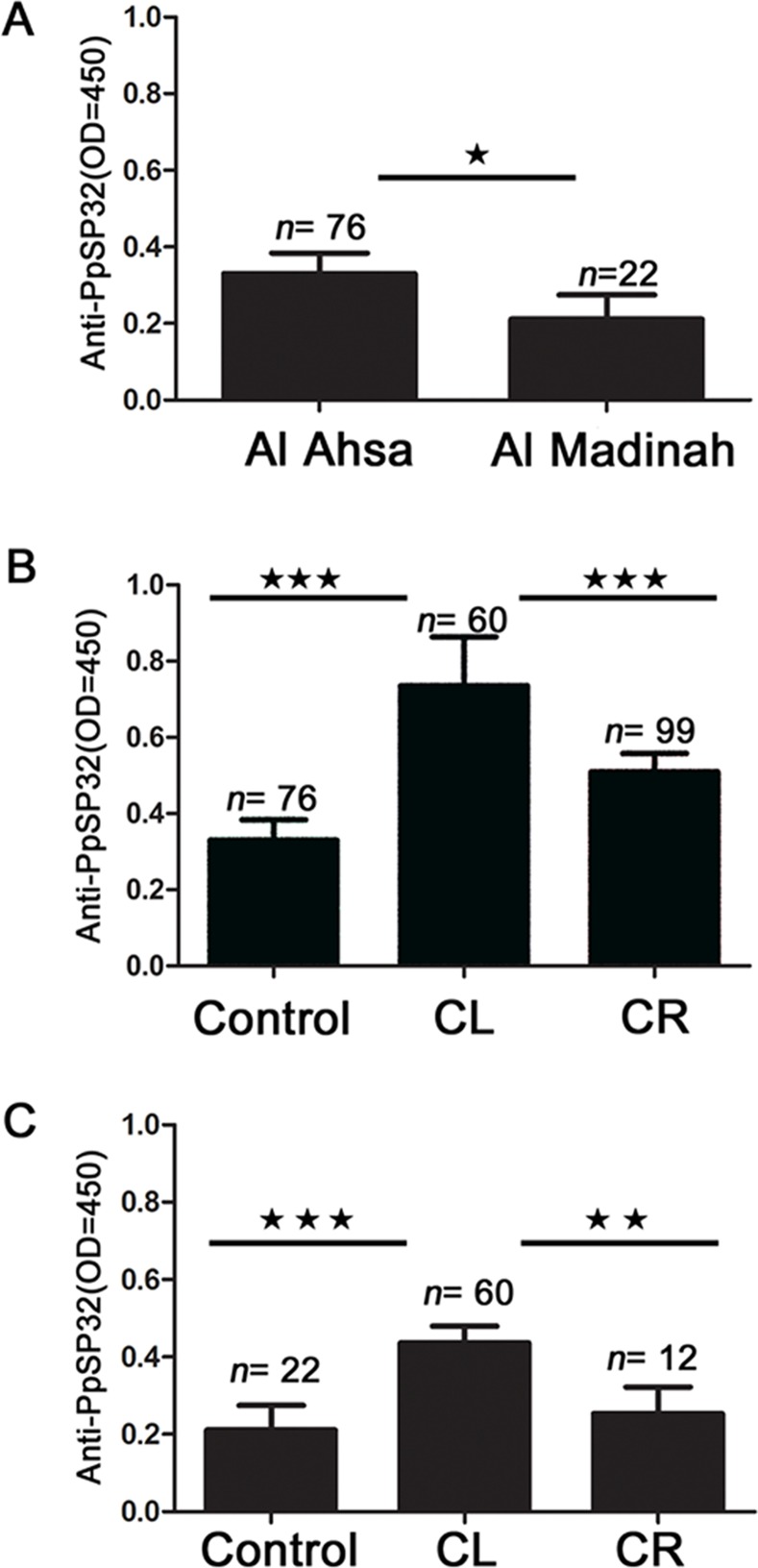
Human antibody response to *Phlebotomus papatasi* salivary protein PpSP32. (**a**) Comparison of anti-PpSP32 antibody levels in healthy individuals from the ZCL regions of Al Ahsa and Al Madinah (**b**) Anti-PpSP32 antibody levels in ZCL patients with active and cured infections from Al Ahsa. (**c**) Anti-PpSP32 antibody levels in ZCL patients with active and cured infections from the region of Al Madinah. Control: healthy individuals; CL: active infection; CR: cured infection; OD: optical density. * *P*≤ .05; ** *P* ≤ .01; *** *P* ≤ .001.

When we compared healthy individuals from the two ZCL endemic regions studied, there was a significantly higher level of anti-PpSP32 antibodies in Al Ahsa compared to Al Madinah (Fig. 2A). To test for a possible correlation between exposure to sandfly bites and leishmaniasis infection, we compared healthy and infected individuals. In both Al Ahsa (Fig. 2B) and Al Madinah (Fig. 2C), patients with an active infection (CL) showed significantly higher levels of anti-PpSP32 antibodies compared to healthy residents (*P*<0.001). Overall, comparing the groups from both Al Ahsa and Al Madinah, the levels of anti-PpSP32 in Al Ahsa individuals appear to be higher than those from Al Madinah, suggesting that Al Ahsa populations are more exposed to *Ph. papatasi* bites.

### PpSP32 is recognized with less extent by individuals living where *Ph. sergenti* is prevalent

**Figure 3:**
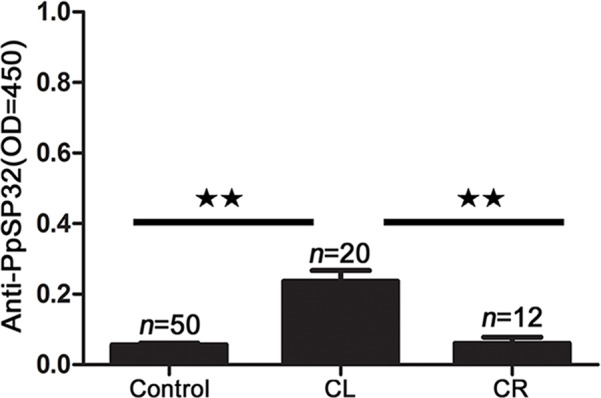
Antibody response to PpSP32 from patients in Asir where *L. tropica* is prevalent. Sera of individuals living in the ACL endemic area of Asir region were tested for anti-PpSP32 antibodies. Control: healthy individuals; CL: active infection; CR: cured infection; OD: optical density. Significance: **P≤ .01.

In individuals from the region of Asir (endemic for ACL *L. tropica* infections), both the healthy and cured groups showed very low levels of anti-PpSP32 antibodies (Fig. 3), which agrees with the near absence of *Ph. papatasi* from this region (Table 1). Unexpectedly, the levels of anti-PpSP32 antibodies were significantly higher (*P*<0.01) in individuals with an active *L. tropica* infection, compared to healthy residents and cured patients (Fig. 3). Sequence alignment of the *Ph. papatasi* PpSP32 [17] and the PpSP32-like protein from *Ph. sergenti* [18] confirmed a significant level of similarity between these homologous proteins (S2 Fig.), suggesting cross-reactivity. Although these patients were Saudi residents and their migration is uncommon, we cannot discard either the possibility that these individuals might have been exposed to *Ph. papatasi* bites while traveling outside this area, or the presence of *Ph. papatasi* in low numbers. In both cases, the anti-PpSP32 levels may reflect a low exposure to this sandfly species.

### Evidence of an association between the levels of anti-PpSP32 antibodies and ZCL clinical presentation

**Figure 4:**
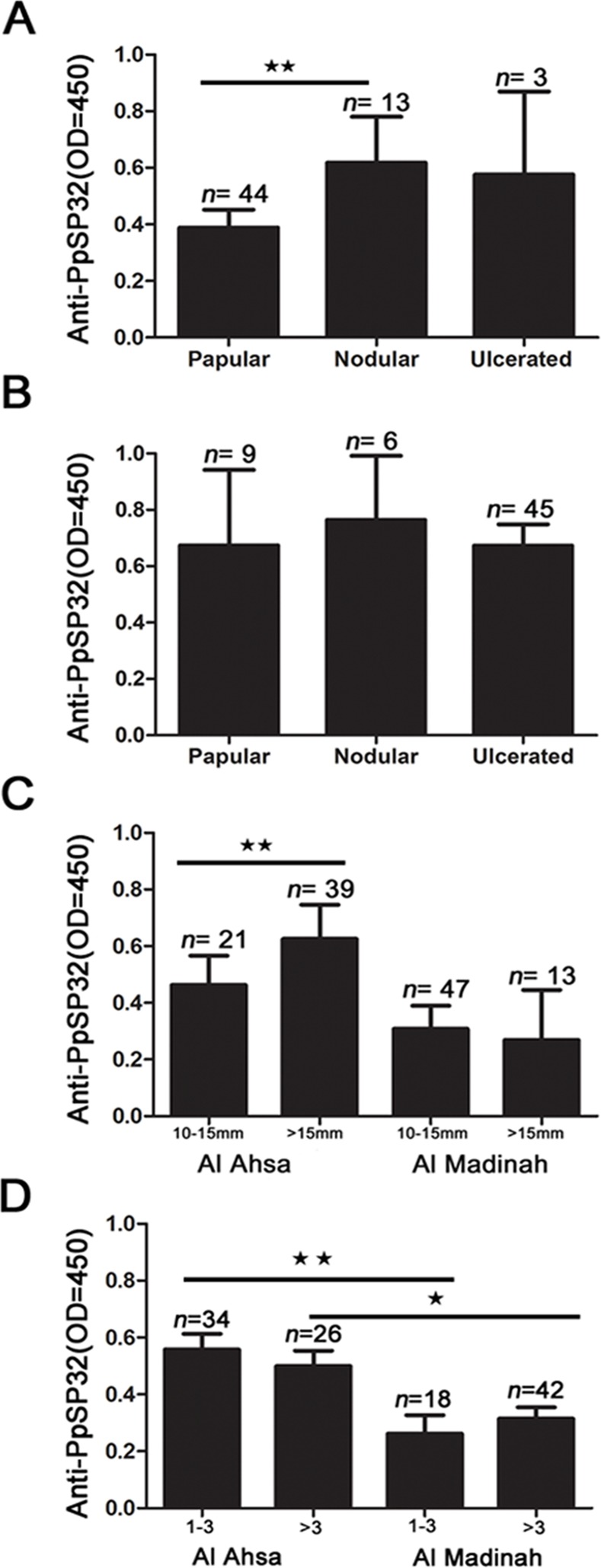
Levels of anti-PpSP32 antibodies in patients with active ZCL vary according to the type and size of the lesions. (**a**) Anti-SP32 antibody levels were measured in patients with nodular, papular and ulcer type lesions in Al Madinah and (**b**) Al Ahsa. (**c**) Comparison of antibody levels according to ZCL lesion size in patients from Al Ahsa (** *p*≤0.01) and Al Madinah. (**d**) Antibody levels according to lesion number in Al Ahsa and Al Madinah. Control: healthy individuals; CL: active infection; CR: cured infection; OD: optical density. * *P*≤ .05; ** *P* ≤ .01; *** *P* ≤ .001.

To test for a correlation between exposure to sandfly bites and the clinical presentations of *L. major* infection in human patients, we compared the levels of anti-PpSP32 antibodies in patients presenting nodular, papular or ulcerated-nodular lesions. Of the three, nodular lesions and then papular are the least severe; both of these lesion types can progress to the more severe ulcerated-nodular form. ZCL patients from Al Madinah with nodular and ulcerated nodular type lesions have higher levels of anti-PpSP32 than those with papular type lesions (Fig. 4A), but a statistical difference was only observed between papular and nodular lesions (*P<0.01*). There were no significant differences in anti-PpSP32 levels between different types of lesions in Al Ahsa patients (Fig. 4B).

We also looked at the levels of anti-PpSP32 in ZCL patients according to the lesion characteristics. Lesion size was classified as being either 10–15mm or >15mm. Patients from Al Ahsa with large lesions >15mm had significantly higher antibody levels (*P*<0.01) than individuals with lesions between 10–15mm (Fig. 4C). This difference was not observed in Al Madinah. Additionally, when we compared the patients with different lesion numbers (< 3 or > 3 lesions) (Fig. 4D), no significant differences in antibody levels were found within each region. However, the same figure shows the difference in anti-PpSP32 levels was significant, with higher levels in Al Ahsa than Al Madinah.

### Visiting labour in Saudi Arabia exhibit a significantly higher antibody response to PpSP32 compared to the residents in Al Ahsa

**Figure 5:**
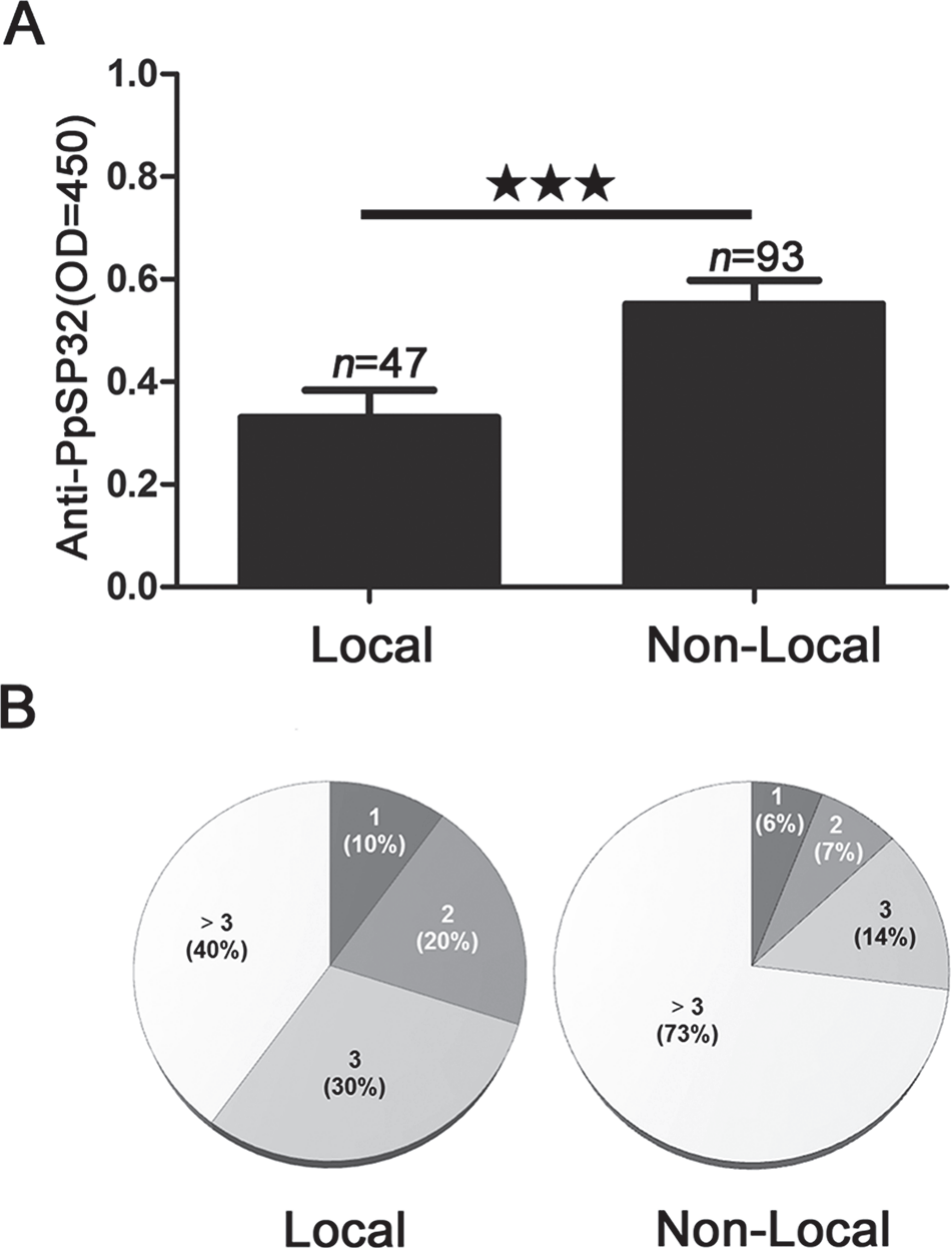
Differential antibody response to PpSP32 between local and non-local patients in Al Ahsa. (**A**) Comparison of anti-PpSP32 antibody levels in local and nonlocal ZCL patients from Al Ahsa. (**B**) Comparison of lesion numbers in local residents and non-local ZCL patients in Al Ahsa. OD: optical density. *** *P* ≤ .001.

In Al Ahsa, we found that non-local patients (visiting labour) had significantly higher levels (*P<0.001*) of anti-PpSP32 compared to the local residents (Fig. 5A). Interestingly, nearly three quarters of the non-local patients developed more than three lesions compared to only 40% in the local group (Fig. 5B). Although such differences did not correlate with the levels of anti-PpSP32 (S3 Fig.), patients from the visiting labour group presented in general a higher number of lesions compared to the residents (S2 Table).

## Discussion

Antibodies to sandfly saliva can be used to indicate disease risk in endemic areas [4,6,12,19], and the development of biomarkers for this purpose depends on the discovery of highly conserved yet species-specific molecules. SP32-like proteins are unique to sandflies and occur in all species studied to date [18]. Among these, PpSP32 is a highly immunogenic protein isolated from the saliva of *Ph. papatasi* that serves as a biomarker for vector exposure [13]. Data obtained from a CL-endemic area in Tunisia showed that the human antibody response to PpSP32 is representative of the humoral response against whole salivary gland extract [6]. Here, we used a recombinant form of this protein to evaluate the level of exposure to sandfly saliva in three endemic areas in Saudi Arabia. Our results show that the severity of human CL pathology appears to be influenced by previous exposure to sandfly bites.

The migration of non-immune people into leishmaniasis endemic areas has been well documented to affect groups such as civilian workers and military personnel [20,21], resulting in leishmaniasis outbreaks [22]. Evaluation of biting exposure can be useful for assessing disease risk of such populations in Saudi Arabia. The higher serum levels of anti-saliva antibodies in the visiting workers compared to the long-term residents of Al Ahsa suggest the migrant population is highly exposed to sandfly bites and less immune to CL. Residents have a lower (but continuous and long-term) exposure to bites, which might induce desensitisation (tolerance) to sandfly saliva, thus explaining their lower antibody levels compared to the non-locals. This desensitization after long term exposure has been previously observed in mice models [23]. Moreover, the residents seem to suffer less severe leishmaniasis lesions. Exposure to uninfected bites of *Ph. papatasi* has been shown to be protective against *L. major* in mice [24] and whether the same level of protection is conferred to humans in CL-endemic areas remains to be determined. Non-locals typically work and dwell closer to sandfly habitats like the burrows of rodents (reservoirs of disease) and are consequently plagued by biting sandflies. Previously unexposed to this level of biting, they showed a more intense antibody response over a shorter period of time. The high exposure to sandfly bites might increase susceptibility to infection and severe clinical outcomes, as nearly three quarters of them developed multiple lesions. Other factors such as genetic background can also influence susceptibility to disease [25]; however, this is unlikely in this situation as the visitors originate from eight different countries, mainly from Middle East, Southern Asia and Africa.

Interestingly, CL patients from both ZCL regions (Al Ahsa and Al Madinah) exhibited even higher levels of anti-PpSP32 antibodies compared to healthy residents from their respective areas. Marzouki et al. [6] previously investigated this relationship using whole salivary gland extract and associated the significantly higher antibody levels in ZCL patients with increased risk of developing CL. This difference was also reported for ACL [12], where exposure to *Ph. sergenti* bites was evaluated in both healthy individuals and patients with *L. tropica*. Similarly, ACL patients produced a significantly higher IgG response compared to healthy people from the same area, likewise supporting the relationship between exposure and leishmaniasis infection. B-cell clonal expansion, which increases production of non-specific antibodies in some parasitic infections [26], could be an alternative explanation to an increased antibody response in CL patients; however, this has only been reported in visceralizing forms of leishmaniasis [27,28].

Our research identified the sandfly species inhabiting the three CL endemic areas in order to complement the data obtained on bite exposure. In agreement with the anti-PpSP32 levels in patient sera, the majority of sandflies found in Al Ahsa and Al Madinah were identified as *Ph. papatasi.* Other sandfly species identified belong to the *Sergentomyia* genus, whose members rarely bite humans (they are mostly zoophilic) and have been shown to be refractory to *Leishmania* species pathogenic to humans [29] *Ph. papatasi* accounts for most, if not all, of the bites sustained by individuals in the ZCL areas. This was further supported by finding significant levels of anti-PpSP32 antibodies in healthy donors of these regions compared to UK control sera. However, anti-PpSP32 antibodies were significantly higher in Al Ahsa, suggesting a higher exposure to *Ph. papatasi* in this region.

Unexpectedly, we found that sera of *L. tropica* patients from the Southwest region of Asir (where *Ph. sergenti* is the predominant CL vector) also recognized PpSP32, although levels were much lower compared to ZCL patients. This could be due to a cross reaction with salivary proteins from *Ph. sergenti*. In fact, there is a high degree of similarity (52%) between *Ph. sergenti* SP32-like protein and *Ph. papatasi* SP32. In mice exposed to *Ph. sergenti* bites, a partial cross-reactivity to *Ph. papatasi* whole salivary gland homogenate was reported [12,30]. A similar level of cross-reactivity could also be present between salivary proteins from *Ph. papatasi* and *Ph. bergeroti* [31] (the second most abundant species in Asir).

Is there a correlation between CL clinical forms and exposure to sandfly bites? We detected higher levels of anti-PpSP32 antibodies in patients with nodular-type lesions compared to those with papular lesions in Al Madinah, but not in Al Ahsa. This differential response could be attributed to a) the genetic background of the infected patients, b) a cumulative exposure to sandfly bites or c) the parasite strains found in each area. It would be interesting to further study how the interaction between these factors affects the immune responses to salivary proteins and disease pathology.

The immune response elicited by sandfly salivary proteins and how it modulates the *Leishmania* infection, varies depending on the vector species and vertebrate host [32]. Some reports have shown that sandfly saliva is able to preferentially trigger a protective Type I delayed-type hypersensitivity response [33–35]. In animal models a Th1 response to salivary proteins is correlated with protection against CL, and immunization with single proteins from sandfly saliva conferred protection against a *L. major* infection when animals were challenged with infectious *Ph. papatasi* bites [35–37]. On the other hand, a Th2 response (and antibodies to salivary proteins) correlates with higher susceptibility and in some cases exacerbation of the disease [38,39]. Furthermore, individuals living in a CL endemic region of Tunisia, where the main vector is *Ph. papatasi*, developed a mixed response with a dominance of Type II immunity [40]. It may possible that subjects that develop antibodies (in a Th2 environment) to PpSP32 (and perhaps other salivary proteins) may be more susceptible to CL. It would be relevant to characterize the immune response(s) in individuals with different clinical presentations and from different geographical locations.

In summary, the use of recombinant salivary proteins can help us understand the impacts of natural exposure to sandflies in leishmaniasis endemic areas [3]. Our results provide insights into the relationship between the human antibody response to sandfly saliva and development of cutaneous leishmaniasis in different transmission contexts. In addition, they support the use of biomarkers as epidemiological tools to improve the surveillance of human-vector contact and disease transmission.

Protein accession numbers (NCBI): *Phlebotomus papatasi* SP32 GI:449060662, *Phlebotomus sergenti* SP44: GI:299829437

## Supporting Information

S1 Checklist STROBE checklist.
(DOCX)

S1 Table Description of the different groups of individuals in the study.
(DOCX)

S2 Table Distribution of local and non-local CL patients from Al Ahsa presenting more than three lesions.
(DOCX)

S1 Fig Levels of anti-PpSP32 antibodies in the sera of healthy individuals from the UK and Saudi Arabia. All the individuals from the ZCL endemic areas in Saudi Arabia exhibited significantly higher levels of antibodies than the non-exposed UK controls (*p*≤0.01).
(DOCX)

S2 Fig Sequence alignment showing similarities between the *Ph. papatasi* and *Ph. sergenti* PpSP32-like proteins. Sequence alignment was carried out using ClustalW and manually annotated. Shading indicates amino acid similarities: Black: fully conserved, Dark Grey: strongly similar; Light Grey: weakly similar.
(DOCX)

S3 Fig Comparison of the antibody levels between locals and non-locals according to the number of lesions. Independent of the number of lesions, visiting workers (non-local patients) showed higher levels of anti-PpSP32 antibodies than the long-term residents (local patients).
(DOCX)
