# Correction: Severity of Old World Cutaneous Leishmaniasis Is Influenced by Previous Exposure to Sandfly Bites in Saudi Arabia

**DOI:** 10.1371/journal.pntd.0004294

**Published:** 2015-12-08

**Authors:** Karina Mondragon-Shem, Waleed S. Al-Salem, Louise Kelly-Hope, Maha Abdeladhim, Mohammed H. Al-Zahrani, Jesus G. Valenzuela, Alvaro Acosta-Serrano

There is an error in the third sentence of the “Detection of human anti-PpSP32 antibodies” sub-section of the Methods section. The concentration of PpSP32 should read 2 ug/ml = 0.2 ug/well, not 2 mg/ml = 0.1 mg/well.
