# Supplementary material for: Severity of Old World Cutaneous Leishmaniasis Is Influenced by Previous Exposure to Sandfly Bites in Saudi Arabia
Source: PLoS Negl Trop Dis. 2015 Feb 3;9(2):e0003449. doi: 10.1371/journal.pntd.0003449 (PMC4315490; doi:10.1371/journal.pntd.0003449)
Supplement: S1 Table — Description of the different groups of individuals in the study. (DOCX) [file pntd.0003449.s002.docx]

**TABLE S1.** Number of participants in each group from the regions of Al Ahsa, Al Madinah and Asir.

| **Region** | | | |
| --- | --- | --- | --- |
|  | **Al Ahsa** | **Al Madinah** | **Asir** |
| **Healthy** | 76 | 22 | 50 |
| **Active Infection (CL)** | 60 | 60 | 20 |
| **Cured (CR)** | 99 | 12 | 12 |
| **Total/region** | 235 | 94 | 82 |
