# Supplementary material for: Severity of Old World Cutaneous Leishmaniasis Is Influenced by Previous Exposure to Sandfly Bites in Saudi Arabia
Source: PLoS Negl Trop Dis. 2015 Feb 3;9(2):e0003449. doi: 10.1371/journal.pntd.0003449 (PMC4315490; doi:10.1371/journal.pntd.0003449)
Supplement: S2 Table — Distribution of local and non-local CL patients from Al Ahsa presenting more than three lesions. (DOCX) [file pntd.0003449.s003.docx]

**TABLE S2.** Distribution of local and non-local CL patients from Al Ahsa presenting more than three lesions.

| **Number of lesions** | | | | | |
| --- | --- | --- | --- | --- | --- |
|  | **4-5** | **6-8** | **9-12** | **13-20** | **20-29** |
| **Local** | 14 (74%) | 3 (16%) | 2 (10%) | 0 (0%) | 0 (0%) |
| **Non-Local** | 22 (32%) | 21 (31%) | 12 (18%) | 7 (10%) | 6 (9%) |
