# Supplementary material for: Severity of Old World Cutaneous Leishmaniasis Is Influenced by Previous Exposure to Sandfly Bites in Saudi Arabia
Source: PLoS Negl Trop Dis. 2015 Feb 3;9(2):e0003449. doi: 10.1371/journal.pntd.0003449 (PMC4315490; doi:10.1371/journal.pntd.0003449)

**Figure S1.** **Levels of anti-PpSP32 antibodies in the sera of healthy individuals from the UK and Saudi Arabia.** All the individuals from the ZCL endemic areas in Saudi Arabia exhibited significantly higher levels of antibodies than the non-exposed UK controls (*p*≤0.01).


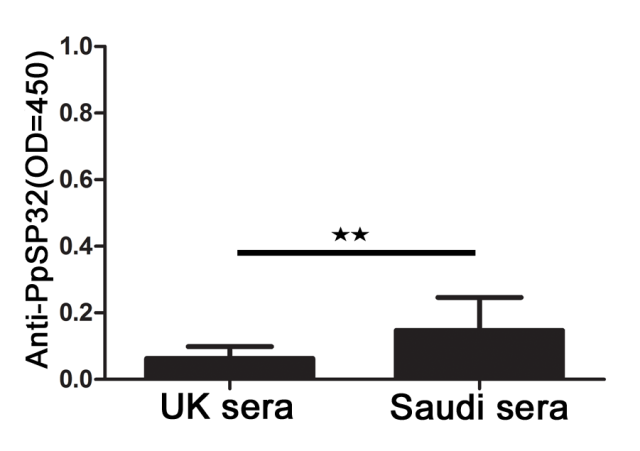

Supplement: S1 Fig — All the individuals from the ZCL endemic areas in Saudi Arabia exhibited significantly higher levels of antibodies than the non-exposed UK controls (p≤0.01). (DOCX) [file pntd.0003449.s004.docx]
