# Supplementary material for: Severity of Old World Cutaneous Leishmaniasis Is Influenced by Previous Exposure to Sandfly Bites in Saudi Arabia
Source: PLoS Negl Trop Dis. 2015 Feb 3;9(2):e0003449. doi: 10.1371/journal.pntd.0003449 (PMC4315490; doi:10.1371/journal.pntd.0003449)
Supplement: S2 Fig — Sequence alignment was carried out using ClustalW and manually annotated. Shading indicates amino acid similarities: Black: fully conserved, Dark Grey: strongly similar; Light Grey: weakly similar. (DOCX) [file pntd.0003449.s005.docx]

**Figure 2S**. **Sequence alignment showing similarities between the *Ph. papatasi* and *Ph. sergenti* PpSP32-like proteins.** Sequence alignment was carried out using ClustalW and manually annotated. Shading indicates amino acid similarities: Black: fully conserved, Dark Grey: strongly similar; Light Grey: weakly similar.
