# Supplementary material for: Severity of Old World Cutaneous Leishmaniasis Is Influenced by Previous Exposure to Sandfly Bites in Saudi Arabia
Source: PLoS Negl Trop Dis. 2015 Feb 3;9(2):e0003449. doi: 10.1371/journal.pntd.0003449 (PMC4315490; doi:10.1371/journal.pntd.0003449)

**Figure S3.** **Comparison of the antibody levels between locals and non-locals according to the number of lesions.** Independent of the number of lesions, visiting workers (non-local patients) showed higher levels of anti-PpSP32 antibodies than the long-term residents (local patients).


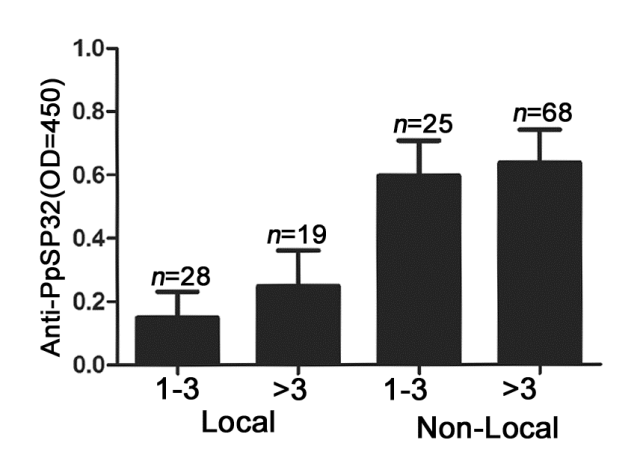

Supplement: S3 Fig — Independent of the number of lesions, visiting workers (non-local patients) showed higher levels of anti-PpSP32 antibodies than the long-term residents (local patients). (DOCX) [file pntd.0003449.s006.docx]
